# Rabies prevention practices and associated factors among household heads in Bure Zuria district, North West Ethiopia

**DOI:** 10.1038/s41598-022-10863-z

**Published:** 2022-05-05

**Authors:** Gedamu Ayalew Wolelaw, Worku Awoke Yalew, Abebaw Gedef Azene, Gizachew Tadesse Wassie

**Affiliations:** Department of Epidemiology and Biostatistics, College of Medicine and Health Science, Bahir Dar University, Bahir Dar, Ethiopia

**Keywords:** Diseases, Health care

## Abstract

Rabies is a zoonotic viral disease that can occur in all warm blooded animals including humans. Vaccinating dogs can protect people from contracting rabies. Despite the availability of effective human and animal rabies vaccines, rabies prevention and control efforts are inadequate. The aim of the study was to determine the level of rabies prevention practices and associated factors among household heads in Bure Zuria district, North-west Ethiopia. Community based cross-sectional study was conducted at Bure Zuria from June 1 to 30, 2020. A total of 609 participants were selected using simple random sampling technique. Simple and multiple binary logistic regressions were applied to identify associated factors of rabies prevention practices. Of 609 participants, 413 (67.8%) were male and 289 (47.5%) were 30–45 years old. The level of good prevention practices of rabies at Bure Zuria district was 43.3%. Being males (AOR = 2.69 (1.72–4.22)), age group 18–29 years (AOR = 2.70 (1.20–6.10)), ever bitten by dog, (AOR = 2.40 (1.56–3.68)), got training (AOR = 1.70 (1.08–2.68)), had dog (AOR = 2.92 (1.62–5.26)), with good knowledge AOR (95% CI) = 3.42 (2.19–5.32), with good attitude AOR (95% CI) = 1.78 (1.16–2.73) and have 1001–2000 AOR (95% CI) = 2.29 (1.39–3.79) and > 2000 AOR (95% CI) = 2.02 (1.28–3.18)) monthly income were more likely to have good prevention practices of rabies. In this study, we found that the level of good prevention practices of rabies was low in Bure Zuria district. Therefore; awareness creation trainings and multi-sectoral collaborations to prevent rabies are needed in the district, zone and at large region level.

## Introduction

Rabies is a zoonotic disease caused by a lyssavirus in the family *Rhabdoviridae*. Carnivorous such as dogs, cats, foxes, jackals, bats, raccoons and skunks are rabies reservoirs depending on the continents ad transmitted to human through close contact with saliva^1,2^. In developing countries, 99% of rabies transmissions to humans are from dogs^3^. Rabies disease is a fatal neglected viral zoonosis which results encephalitis in many warm blooded animals and humans^4^. The incubation period of rabies varies from five days to several years depending on the proximity of virus entry to the central nervous system and the amount of virus in the inoculum^2,5^.

It is preventable disease through vaccination. However; the burden of rabies mortality in developing countries was high due to inadequate prevention practices and unavailability of vaccines^6,7^. It constitutes a serious public and animal health problem globally which causes over 60,000 human deaths per year^3^. More than 95% of deaths occur in Africa and Asia. Of these, 44% of deaths accounted in Africa^8^. It has also yearly cost of estimated US$ 583.5 million in Asia and Africa most of which is due to post exposure prophylaxis (PEP) expenses^9^.

In Ethiopia, rabies has been known for hundreds of years in society as “Mad Dog illness” and over 2,700 human die due to rabies annually^10,11^. Individuals who baited by suspected dog often prefer traditional healers for the diagnosis and treatment of the disease. These widespread traditional practices; results delayed modern medical care^12–14^. The actual magnitude of the problem is not well known in Ethiopia. Besides the distribution of anti-rabies vaccine is not adequate^15^.

Rabies disease prevention practices are attributed by high rate of unvaccinated dogs, lack of awareness about rabies vaccination among dog owners, residence, knowledge, attitudes and practices (KAP) as evidenced from previously done studies^1,2,16–18^. Due to low rabies vaccination coverage among dogs in Ethiopia, the number of new human rabies exposure cases increased from 35.8, in 2012 to 73.1 in 2015 per 100,000 populations^4^. Of which human rabies exposure cases 664 (71.9%) were from rural settings. Dogs were the principal sources of exposure (96.3%)^3,12^.

It is well known that rabies disease if preventable. However, no study was conducted in the study area. Therefore; this study aimed to assess the level of rabies prevention practices and its associated factors among the residents of Bure Zuria district, west Gojjam, North west Ethiopia.

## Materials and methods

### Study area

**Figure 1:**
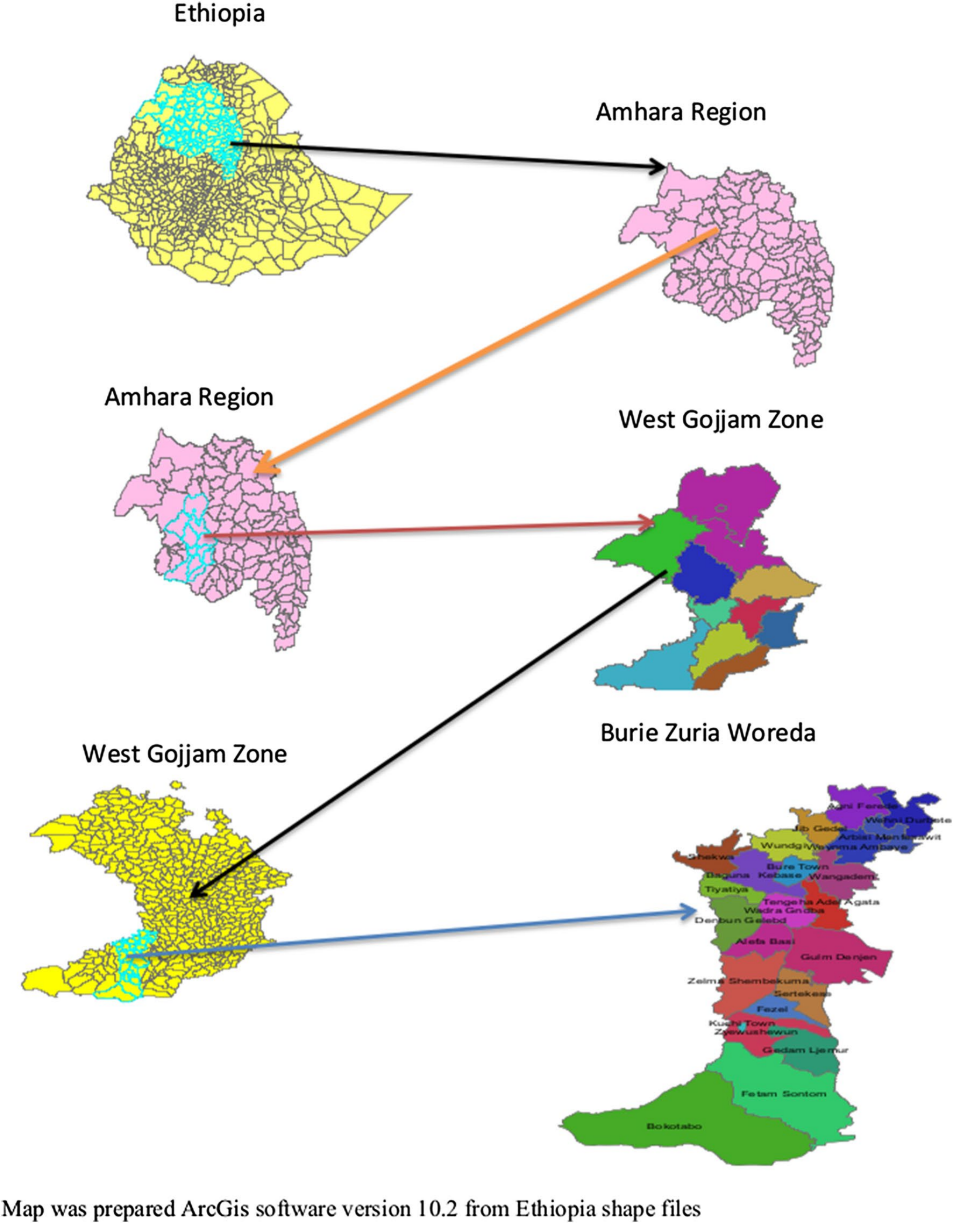
Map of Bure Zuria district which prepared ArcGis software version 10.2 from Ethiopia shape files.

This study area was Bure Zuria district, North West Ethiopia. Bure Zuria is one of the woredas in the Amhara Region of Ethiopia, Part of the West Gojjam Zone, 408.7 km Northwest of Addis Ababa and 150 km from Bahir Dar. Bure Zuria is bordered on the south by the Abay River which separates it from the Oromia Region, on the west by Womberma, on the northwest by the Agew Awi Zone, on the north by Sekela, on the east by Jabi Tehnan, and on the southeast by Dembecha and the Misraq Gojjam Zone. Bure Zuria was part of former Bure woreda. According to the projected 2007 national census conducted by the Central Statistical Agency of Ethiopia (CSA), the district has a total human population of 131, 817 (112,248 were males and 110,129 were females). Ninety percent of the population in the district is rural inhabitants. The district comprises 19 ‘Kebeles’ and the study were conducted in 6 of them. The majority of the inhabitants practiced Ethiopian Orthodox Christianity, with 98.34% reporting that as their religion, while 1.01% was Muslim (Fig. 1).

### Study design and period

A community based cross-sectional study design was conducted from June 1 to 30, 2020.

### Source population

All household heads who live in Bure Zuria district were the source population for this study.

### Study population

The study population was household heads who live in randomly selected kebeles of Bure Zuria district (Wadra, wohine, Z/shenu, gulum, Arbisie and Woynema ambaye) as permanent residents for more than six months.

### Eligible criteria

#### Inclusion criteria

Household heads that have lived more than 6 months as a permanent resident in the study area were included.

#### Exclusion criteria

Respondents in the household who cannot communicate and under18 year were excluded from this study.

### Sample size determination, sampling techniques, and procedure

#### Sample size determination

The required sample size for this study was estimated by considering 60% of the population had good practices scores towards rabies prevention from earlier study conducted^19^. Sample size was determined using Cochran’s sample size formula for categorical data. The sample size was calculated using single population proportion formula with 5% margin of error, 95% confidence level, 1.5 design effect, and 10% none response rate then the final calculated sample size for prevention practices towards rabies was 609.

#### Sampling techniques and procedure

The calculated sample was proportionally distributed to the selected kebele based on their number of households. A multi-stage random sampling technique was employed for the selection of the sampling units. Out of 19 ‘Kebeles’ in the district, 6 of them were selected by simple random sampling methods. Then, the household in the selected kebeles were further selected using a systematic random sampling technique. From each household, a person who economically supports or manages the household (heads of the family) was interviewed. However, in the absence of eligible respondents in a given household, a replacement was immediately made by an individual in the next household until required sample size obtained.

### Variables of the study

**Dependent variable:** level of rabies prevention and control practices (good and poor).

#### Independent variables

**Socio-demographic factors**: sex, Age, educational status, occupation, family size, marital status and monthly income.

**Personal factor**: history of dog bite to family members, training status, dog ownership status and source of information.

**Knowledge:** good and poor.

**Attitude:** positive and negative.

#### Operational definition

**Good practices score**: respondents who scored points at mean and above for the Practices questions prepared were referred to be having good Practices score.

**Poor practices score**: respondents who scored points below mean for the Practices questions prepared were referred to be having poor Practices score.

**Good knowledge**: respondents who scored points at mean and above for the knowledge questions prepared were referred to be having good knowledge otherwise not.

**Positive attitude**: respondents who scored points at mean and above for the attitude questions were referred to be having positive attitude otherwise not.

### Data collection instruments and procedure

Data was collected using 30 pre-tested and structured Amharic version questionnaires of face to face interview for the socio-demographic characteristic, Knowledge, Attitude and Practices questions about rabies. Four nurse health workers who are diploma graduates and one health officer as supervisor were recruited to collect the data. Before beginning data collection, for two day adequate training was given to the data collectors about rabies, the objective of standardizing the data collection instrument among the data collectors, basic skill of communicating with the study participants and data collection procedures. Primary data on sociodemographic characteristics of households, nine knowledge questions on rabies (description of the disease, mode of transmission, outcome, sign and symptoms, range of species affected and means of prevention and control), six attitude questions and eight practices questions towards rabies prevention and control. The questions were multiple choice and yes and no questions were included. Participants who answered the questions correctly had got one mark and zero for incorrect or do not know responses. Then, the responses for which respondents give correct answer was counted and scored. This score was pooled together and the mean score was computed to determine knowledge, attitude and practices of Participants. Respondents who score greater than or equal to the mean value (Mean = 5.79, SD = 1.22) grouped to good knowledge and less than the mean value poor knowledge level, respondents who score greater than or equal to the mean value (Mean = 3.62, SD = 1.48) grouped to positive attitude and less than the mean value negative attitude level, respondents who score greater than or equal to the mean value (Mean = 3.47, SD = 1.6) grouped to good practices and less than the mean value poor practices level.

### Data quality control

The questionnaire was adjusted and modified in to our context from previous literatures. The questionnaire, originally prepared in English was translated to the local language, Amharic for appropriateness and easiness in approaching the study participants and then retranslated back to English by an expert who is fluent in both languages to maintain its consistency. This questionnaire was administered to 5% of sample size randomly selected individuals outside of the study area, in Jabitehna district to check the understandability comprehension of the questions, and simultaneously it was used as part of the data collection training for data collectors. Ambiguous words were made clearer based on the feedback.

The data was collected through face-to-face interviews. After the data collection process principal investigator again checked the collected data for completeness or misfiled questionnaire, clarity, consistency and accuracy on daily basis. Then questionnaires were cleaned and coded for computer data entry by principal investigator. The data was then analyzed using SPSS statistical software version 23.

### Statistical analysis

The analyses were started by data entering, coding and cleaning. Data entry was carried out using the EPI INFO version 7software. After completion of data entry, recorded data was exported to SPSS virsion-25 and analyzed. The frequency distribution of both dependent and independent variable were done. Variables which have p-value ≤ 0.2 in simple binary logistics regression model were included in multiple binary logistics regression to identify the associated factors. A 95% confidence interval of the OR and p-values < 0.05 was used to describe statistical significance, strength, and direction of association.

### Ethical approval and consent to participate

Ethical clearance was obtained from Institutional Review Board of Institute of Public Health, College of Medicine and Health Sciences, Bahir Dar University and official written informed consent was obtained from Amhara Regional Health Bureau/ Regional Health Research Laboratory Center, West Gojjam Zonal Health Department and Bure Zuria district Administration Health Office for official start of the study. Letters was also prepared to the local authority of the selected kebeles, health centers head and health posts by the Woreda health office. The study was conducted in strict accordance with the ethical standards set forth in the 1964 Declaration of Helsinki and the ethical review board of Public Health, College of Medicine and Health Sciences, Bahir Dar University, Ethiopia. After informed the purposes and importance of the study, written informed consents were taken from all household heads.

## Results

### Socio-demographic and economic factors

A total of 609 household heads were interviewed in this research, which results a response rate of 100%. More than half 413 (67.8%) of the interviewed participants were males. Regarding age group, the majority 289 (47.5%) of participants age were between 30–45 years old. From all participants of the study, 343 (56.3%) were married. Regarding the religion, all respondents 609 (100%) was orthodox. About the educational status of respondents, 529 (86.9%) were unable to read and write. Concerning the household size, about 237 (38.9%) participants were from family size of above six persons and also most of the respondents, 599 (98.4%) were farmers (Table 1).

**Table 1:** Socio-demographic and economic factors of study participants.

Variables	Frequency (%)
**Sex**	
Male	413 (67.8%)
Female	196 (32.2%)
**Age in years**	
18–29	54 (8.9%)
30–45	289 (47.5%)
> 45	266 (43.7%)
**Marital status**	
Married	343 (56.3%)
Unmarried	74 (12.2%)
Divorced	120 (19.7%)
Widowed	58 (9.5%)
Separated	14 (2.3%)
**Occupation**	
Farmer	599 (98.4%)
Merchant	10 (1.6%)
**Educational status**	
Unable to read and write	529 (86.9%)
Elementary	70 (11.5%)
High school	10 (1.6%)
**Economic status**	
< = 1000	237 (38.9%)
1001–2000	158 (25.9%)
> 2000	214 (35.1%)
**Household size**	
1–3	168 (27.6%)
4–6	204 (33.5%)
> 6	237 (38.9%)

From the whole participants, about 523 (85.9%) had dogs. About 149 (24.5%) of respondents, obtained information about rabies from health professionals. The overall mean scores of respondent’s knowledge on rabies disease was 5.79 (± 1.22). About 349 (57.3%) of participants were above the mean score level as having good knowledge on rabies prevention and control. And also the overall mean attitude score of respondents were 3.62 (± 1.48) in this study. Around half 312 (51.2%) of participants were above the mean score level; had good attitude towards rabies prevention and control. From the total participants, 410 (67.3%) participants and their family members were ever bitten by a dog (Table 2).

**Table 2:** The personal factors, knowledge and attitude of study participants towards rabies prevention and control practices in Bure Zuria District, North West Ethiopia.

Variables	Frequency (%)
**Ever bitten by dog**	
No	199 (32.7%)
Yes	410 (67.3%)
**Ever got training**	
No	454 (74.5%)
Yes	155 (25.5%)
**Dog ownership**	
No	86 (14.1%)
Yes	523 (85.9%)
**Source of information**	
Health professionals	149 (24.5%)
Friends	66 (10.8%)
Neighbors	28 (4.6%)
no information	95 (15.6%)
Radio	116 (19%)
Relatives	43 (7.1%)
Traditional healer	112 (18.4%)
**Knowledge**	
Good	349 (57.3%)
Poor	260 (42.7%)
**Attitude**	
Good	312 (51.2%)
Poor	297 (48.8%)

### Community practices regarding rabies prevention and control

In this study 66 (10.8%) of participants control their dogs in secure cage. From the total 523 dog owners, 155 (29.6%) had vaccinated their dog, the rest 368 (70.6%) of the dog owners did not vaccinate their dog. The reason behind not vaccinating was; 108 (29.3%) did not have awareness, 104 (28.3%) believed that vaccine was unreliable due to unreliable on efficacy of vaccine. About actions to be taken for a suspected rabid animal, 487 (80%) of the participants preferred killing the animal.

Regarding rabid animal meat; 424 (69.6%) of the study participants eat rabid animal meat. Concerning action taken for bitten human, go to health facility or vaccination was responded by 122 (20%) of the participants. Regarding post exposure prophylaxis of the respondents 123 (20.2%) preferred immediately.

Generally the finding of this study showed that (43.3%) of the study participants were found to have overall good Practices scores about rabies prevention and control (Table 3).

**Table 3:** Rabies prevention practices among household heads in Bure Zuria district, North Ethiopia, Jun 01–30/2020.

Variables	Frequency (%)
**How do you control your dog**	
In secure cage	66 (10.8%)
Tie in compound	168 (27.6%)
Lie free	375 (61.6%)
**How do you control stray dog**	
Killing	182 (29.9%)
Contact owners	244 (40.1%)
Inform to authority	183 (30%)
**Action after dog bite**	
Washing with water and soap	121 (19.9%)
Go to health facility or vaccination	122 (20%)
Traditional healer	242 (39.7%)
Nothing to be done	124 (20.4%)
**Timing of post exposure prophylaxis**	
Immediately	123 (20.2%)
Later (02–14 day)	121 (19.9%)
Any time (> 14 day)	151 (24.8%)
Do not know	214 (35.1%)
**Do you eat rabid animal meat**	
Yes	424 (69.6%)
No	185 (30.4%)
**Ever vaccinated your dog**	
Yes	155 (29.6%)
No	368 (70.4%)
**Action taken to rabid dog**	
Do nothing	67 (11%)
Restrain	55 (9%)
Killing	487 (80%)
**Inform to authority if you bitten by rabid dog**	
Yes	243 (39.9%)
No	366 (60.1%)
**Why not vaccinated**	
No awareness	108 (29.3%)
Cost of the vaccine	54 (14.7%)
Unreliable on efficacy	102 (27.7%)
Vaccine inaccessibility	104 (28.3%)
**Practices score**	
Good score (≥ mean score)	264 (43.3%)
Poor (< mean score)	345 (56.7%)

### Factors associated with rabies prevention practices

In simple and multiple binary logistic regression analysis, different variables were significantly associated with the prevention practices score level of the study participants regarding rabies. Variables such as dog ownership, marital status, educational status, ever got training, ever bitten by dog, source of information, sex of respondent, age of respondent, monthly income, knowledge and attitude were associated prevention practices of rabies in the simple binary logistics regression.

In multiple binary logistics regression analysis resulted that dog ownership, ever got training, ever bitten by dog, source of information, sex of respondent, age of respondent, monthly income, knowledge had statistically significant association with practices about rabies at 5% level of significance. Males were 2.69 times more likely to have good prevention practices towards rabies than females (AOR = 2.69; 95% CI 1.72–4.22)). An individual whose age group was 18–29 years were 2.7 times more likely to had good prevention practices towards rabies than whose age group greater than 45 years (AOR = 2.70; 95% CI 1.20–6.10)). The association of dog ownership level with prevention practices score showed statically significant difference (P = 0.000). The odds of participants who had dog were 2.92 times more likely to have good prevention practices than those who did not have dog (AOR = 2.92; 95% CI 1.62–5.26)). The variables taking training on rabies (AOR = 1.70; 95% CI 1.08–2.68)), having good knowledge (AOR = 3.42; 95% CI 2.19–5.32), good attitude (AOR = 1.78; 95% CI 1.16–2.73)) , Respondents with relative (AOR = 0.20; 95% CI (0.08–0.49)), neighbors (AOR = 0.16; 95% CI (0.05–0.45)), friends (AOR = 0.27; 95% CI (0.131–0.56)), radio as source of information (AOR = 0.49; 95% CI (0.27–0.87)), and monthly income with 1001–2000 (AOR = 2.29; 95% CI (1.39–3.79)) and > 2000 (AOR = 2.02; 95% CI (1.28–3.18)) were significantly associated with prevention practices of rabies (See Table 4).

**Table 4:** Factors associated with good rabies prevention practices among household head in Bure Zuria district, Amhara, Ethiopia, Jun 01–30/2020.

Variables	Prevention practices rabies			
	Good	Poor	COR (95%CI)	AOR (95%CI)
**Do you have dog**				
No	24 (27.9%)	62 (72.1%)	1	1
Yes	240 (45.9%)	283 (54.1%)	2.19 (1.33–3.62)	2.92 (1.62–5.26)
**Source of information**				
Health professionals	88 (59.1%)	61 (40.9%)	1	1
Friends	21 (31.8%)	45 (68.2%)	0.32 (0.18–0.60)	0.27 (0.132–0.56)
Neighbors	7 (25%)	21 (75%)	0.23 (0.092–0.58)	0.16 (0.05–0.45)
No information	38 (40%)	57 (60%)	0.46 (0.27–0.78)*	0.92 (0.48–1.77)
Radio	51 (44%)	65 (56%)	0.54 (0.33–0.89)	0.49 (0.27–0.87)
Relatives	10 (23.3%)	33 (76.7%)	0.21 (0.096–0.46)	0.20 (0.08–0.49)
Traditional healer	49 (43.8%)	63 (56.2%)	0.54 (0.33–0.89)	0.56 (0.31–1.004)
**Marital status**				
Married	158 (46.1%)	185 (53.9%)	1	1
Unmarried	26 (35.1%)	48 (64.9%)	0.63 (0.38–1.07)*	0.54 (0.28–1.04)
Divorced	55 (45.8%)	65 (54.2%)	0.99 (0.65–1.50)	1.64 (0.96–2.80)
Widowed	19 (32.8%)	39 (67.2%)	0.57 (0.32–1.03)*	0.96 (0.469–1.96)
Separated	6 (42.9%)	8 (57.1%)	0.88 (0.30–2.59)	0.74 (0.23–2.44)
**Sex**				
Female	53 (27%)	143 (73%)	1	
Male	211 (51.1%)	202 (48.9%)	2.82 (1.95–4.08)	2.69 (1.72–4.22)
**Have you ever bitten by dog**				
No	54 (27.1%)	145 (72.9%)	1	1
Yes	210 (51.2%)	200 (48.8%)	2.82 (1.95–4.07)	2.40 (1.56–3.68)
**Ever got training about rabies**				
No	177 (39%)	277 (61%)	1	1
Yes	87 (56.1%)	68 (43.9%)	2.00 (1.38–2.90)	1.70 (1.08–2.68)
**Attitude score cat**				
Good	164 (52.6%)	148 (47.4%)	2.18 (1.57–3.03)	1.78 (1.16–2.73)
Poor	100 (33.7%)	197 (66.3%)	1	1
**Knowledge cat**				
Good	197 (56.4%)	152 (43.6%)	3.73 (2.63–5.29)	3.42 (2.19–5.32)
Poor	67 (25.8%)	193 (74.2%)	1	1
**Age cat**				
18–29	35 (64.8%)	19 (35.2%)	2.65 (1.44–4.88)	2.70 (1.20–6.10)
30–45	120 (41.5%)	169 (58.5%)	1.02 (0.73–1.43)	1.05 (0.68–1.62)
> 45	109 (41%)	157 (59%)	1	1
**Monthly income cat**				
< = 1000	73 (30.8%)	164 (69.2%)	1	1
1001–2000	74 (46.8%)	84 (53.2%)	1.98 (1.305–3.00)	2.29 (1.39–3.79)
> 2000	117 (54.7%)	97 (45.3%)	2.71 (1.84–3.98)	2.02 (1.28–3.18)
**Educational status**				
Elementary	34 (48.6%)	36 (51.4%)	1.30 (0.79–2.14)	1.15 (0.59–2.24)
High school	7 (70%)	3 (30%)	3.20 (0.82–12.52)*	1.21 (0.25–5.85)
Unable read and write	223 (42.2%)	306 (57.8%)	1	1

*‘P-value <  = 0.2’ in the bivariate analysis.

## Discussion

Rabies is a major public health problem in Ethiopia. Assessing the level of prevention practices and associated factors towards rabies is important to control the distribution of disease in the community. The distribution of the disease is depending on the socio-demographic and cultural factors. We found in this study that the prevalence of good rabies prevention practices among house hold heads of Bure Zuria district was 43.3% (95% CI 39.4, 47.2). This was higher than the studies done in Laelay–Machew district in 2018 which was 37.6% of the participants had good prevention practices towards rabies and in Bhutan which was 14%^17,20^. This discrepancy might be due to time difference which could bring a difference on awareness of study participants. But this finding was lower than the studies conducted in Mekele city, Grenada and Nigeria which were 61.3%, 51.6%, and 75%, respectively^19,21,22^. This difference might be due to absence of health education programs about rabies in this study area.

Moreover, rabies prevention practices affected by different factors and the findings of this study shows different variables, which were significantly associated with rabies prevention practices. Multiple binary logistics regression analysis revealed that sex was significantly associated with rabies prevention practices scores, those who were male household heads were 2.69 times more likely to have good practices towards rabies prevention than female. This result was in line with KAP study done in Bahir Dar city male (53.4%) and female (10.75%)^12^, Munesa district, Arsie zone male (69.6%) and female (39.3%)^23^ and Debark^7^. This might be due to that male get awareness about rabies from different meeting by rural health extension workers and better chance of acquiring correct information about rabies.

And also multivariable analysis result of this study also revealed that good Practices score was significantly associated with age; those in age group 18–29 years were 2.7 times more likely to have good practices towards rabies prevention and control than those older than 45 years. This might be due to younger individuals read, heard about the disease and how it can be controlled via the media or by discussions with other community members than older individuals. This result was contrary to that with study done in Shirsuphal village older than 35 years of age (OR 2.08)^9^. This might be due to study area difference since this study conducted in rural district in farmers; in which older participants are less likely to be literate than the previous study.

Furthermore, the findings of this study also revealed that the association of monthly income with Practices scores revealed statistically significant difference. The respondent who have 1001–2000 and > 2000 AOR (95% CI) = 2.02 (1.28–3.18)) monthly income were 2.29 times more likely to have good Practices score than respondents with <  = 1000 monthly income. This result was in line with KAP Study conducted in Bahir Dar town^12^. This might be due to those participants in the community with high/middle income have frequent contact with different person including animal health workers while they discuss about the different effective farming methods to improve their economic status, in this time they got information about rabies. In addition to this participants with middle/high income might vaccinate their do irrespective of the cost of the vaccine.

A finding of this study also revealed that household heads who had dogs were 2.92 times more likely to have good Practices scores than those who do not have dog (AOR = 2.92 (1.62–5.26)). This result was in line with studies done in Mekele city and Injibara Town^19,24^. The possible justification for this could be those who have dogs got good information about rabies in the time of vaccination, on how to care dogs and prevent rabies exposure.

Moreover; the results of this study revealed that good practices scores was significantly associated with dog bite history; ever bitten by dog were 2.4 times more likely to have good practices than not ever bitten (AOR = 2.40 (1.56–3.68)). This result was similar with study done in Mekele city 5.25 (2.09, 13.2) and Tanzania^2,19^. The possible reason for this could be those who have ever bitten got good information about rabies in the time of vaccination or wound care, on how to care dogs and prevent rabies exposure.

Furthermore, this study revealed that the association of source of information with Practices scores revealed statistically significant difference, Participants that relatives, neighbors, friends and radio as their sources of information were 0.28 times less likely to have good practices than participants got information from health professionals AOR (95% CI) 0.28 (0.131–0.56). This result was similar with that of the study done in Laelay-Machew district^20^. This might be due to lack of appropriate and comprehensive information on rabies transferred from relatives, neighbors and friends. In addition participants in the community might give less attention to those sources of information than health professionals.

And also, the respondents training status on rabies were significantly associated with good practices score, respondents who got training were 1.7 times more likely to have good practices than non-trained respondents (AOR = 1.70 (1.08–2.68)). This was consistent with KAP study done in dedo district in Jimma zone (AOR = 3.37 (1.17–9.69))^25^. This indicated that giving training and health education is important to increase the awareness of the community which result good rabies prevention and control practices.

And also the multivariable analysis result of this study revealed that the respondents attitude score on rabies were significantly associated with Practices scores, respondents having good attitude were 1.78 times more likely to have good practices than respondents having poor attitude AOR (95% CI) = 1.78 (1.16–2.73).This result was similar with study done in Laelay–Machew district^20^. The possible explanation might be participants who have good attitude, practices the information they got from different source about rabies without negligence.

The respondents knowledge scores on rabies were significantly associated with Practices score, Participants who have good knowledge AOR (95% CI) 3.42 (2.19–5.32) were 3.42 times more likely to have good practices than participants with poor knowledge. This was similar with Study conducted in Sokoto, Nigeria good practices of rabies prevention was significantly higher among respondents with good knowledge of cause and transmission and prevention of rabies (37.3%) as compared to those with poor knowledge (15%)^26^. The possible reason is that knowledge in prevention and control of rabies has important role on prevention and control practices of rabies. Community awareness about rabies has significant role in rabies prevention and control^27^.

Furthermore, no statistically significant difference was observed between practices scores level and marital status educational status of the respondents, occupational status and religion in this study. However, these variables were statically significant in other previous studies done in BahirDar^12^, Sokoto, Nigeria^26^ and Addis Ababa^10^. This might be due to study area difference; since this study area was rural where no educational status and religion difference. And also it might be due to sample size difference.

## Conclusion

This study we found that overall good rabies prevention practices was low in rural Bure Zuria district. The variables Sex, age, dog ownership, training history, ever bitten by dog, source of information, monthly income, knowledge and attitude of the respondents were found significantly associated with prevention practices on rabies. The implications of this findings that further awareness creation activities and multi-sectoral collaborations to prevent rabies are needed in the district, zone and at large region. Furthermore, Ethiopian public health emergence management institute should increase the availability and distribution of vaccine in different health facilities.

## Data Availability

All data will be accessible form the correspondence author for a reasonable request.

## Abbreviations

AOC: Adjusted odds ratio
KAP: Knowledge, attitude and practices
CSA: Central statistical agency
PEP: Post exposure prophylaxis
WHO: World Health Organization
